# MyBack - effectiveness and implementation of a behavior change informed exercise programme to prevent low back pain recurrences: a hybrid effectiveness-implementation randomized controlled study protocol

**DOI:** 10.1186/s12891-024-07542-7

**Published:** 2024-06-05

**Authors:** Diogo Pires, Susana Duarte, Ana Maria Rodrigues, Carmen Caeiro, Helena Canhão, Jaime Branco, Joana Alves, Marta Marques, Pedro Aguiar, Rita Fernandes, Rute Dinis Sousa, Eduardo B. Cruz

**Affiliations:** 1 Instituto Politécnico de Setúbal, Escola Superior de Saúde, Setúbal, Portugal; 2 Comprehensive Health Research Centre, NOVA Medical School, Universidade Nova de Lisboa, Lisboa, Portugal; 3 NOVA National School of Public Health, Public Health Research Centre, Comprehensive Health Research Center, CHRC, NOVA University Lisbon, Lisboa, Portugal; 4 EpiDoC Unit, NOVA Medical School, NOVA University of Lisbon, Lisboa, Portugal; 5 Unidade de Reumatologia, Hospital dos Lusíadas, Lisboa, Portugal; 6 Unidade de Reumatologia, CHULC Hospital Santo António dos Capuchos, Lisboa, Portugal; 7 Serviço de Reumatologia do Hospital Egas Moniz—Centro Hospitalar Lisboa Ocidental, Lisboa, Portugal; 8 LBMF, CIPER, Faculdade de Motricidade Humana, Universidade de Lisboa, Cruz-Quebrada, Dafundo, Portugal

**Keywords:** Low back pain, Recurrences, Exercise, Behaviour change, Hybrid type I implementation/ effectiveness, Primary health care

## Abstract

**Background:**

Low back pain (LBP) is a common health condition and the leading cause of years lived with disability worldwide. Most LBP episodes have a favourable prognosis, but recurrences within a year are common. Despite the individual and societal impact related to LBP recurrences, there is limited evidence on effective strategies for secondary prevention of LBP and successful implementation of intervention programmes in a real-world context. The aim of this study is to analyse the effectiveness of a tailored exercise and behavioural change programme (MyBack programme) in the secondary prevention of LBP; and evaluate acceptability, feasibility and determinants of implementation by the different stakeholders, as well as the implementation strategy of the MyBack programme in real context.

**Methods:**

This protocol describes a hybrid type I, randomized controlled trial to evaluate the effectiveness and implementation of MyBack programme in the context of primary health care. The Behaviour Change Wheel framework and FITT-VP principles will inform the development of the behaviour change and exercise component of MyBack programme, respectively. Patients who have recently recovered from an episode of non-specific LBP will be randomly assigned to MyBack and usual care group or usual care group. The primary outcome will be the risk of LBP recurrence. The secondary outcomes will include disability, pain intensity, musculoskeletal health, and health-related quality of life. Participants will be followed monthly for 1 year. Costs data related to health care use and the MyBack programme will be also collected. Implementation outcomes will be assessed in parallel with the effectiveness study using qualitative methods (focus groups with participants and health providers) and quantitative data (study enrolment and participation data; participants adherence).

**Discussion:**

To our knowledge, this is the first study assessing the effectiveness and implementation of a tailored exercise and behaviour change programme for prevention of LBP recurrences. Despite challenges related to hybrid design, it is expected that data on the effectiveness, cost-effectiveness, and implementation of the MyBack programme may contribute to improve health care in patients at risk of LBP recurrences, contributing to direct and indirect costs reduction for patients and the health system.

**Trial registration number:**

NCT05841732.

## Highlights

• LBP recurrences are common and account for a significant proportion of the costs and disability associated with low back pain. However, evidence on secondary prevention of LBP remains scarce.
• A hybrid effectiveness-implementations design will be used to analyse the effectiveness, cost-effectiveness, and implementation of an exercise and behavior change programme in primary care.
• Mixed method will used to analyze the implementation strategies. This data could inform the refinement of the implementation strategy and promote the adoption of the MyBack programme in the future.
• This study data may improve the delivery of care in people at risk of recurrence and bring important benefits to individuals and society.

## Background

Low back pain (LBP) is the most prevalent musculoskeletal health condition worldwide, affecting about 568 million people of all social classes and age groups [1]. According to Global Burden of Disease study 2019, LBP remains the leading cause of years lived with disability (YLDs), accounting for 63.7 million YLDs (an increase of 47% since 1990) [1, 2]. In Portugal, the estimated point prevalence of LBP was 26.4% [3] and recent national projections suggest an increase of more than 8% by 2050 [4]. LBP has been consistently associated with long-term disability, anxiety and depressive symptoms, and high costs associated with loss of working hours and early retirement [3, 5]. LBP episodes are one of the main reasons for consultation in primary health care [6, 7]. The levels of disability due LBP are responsible for most of the healthcare costs associated with treatment, including high medicalization rates, imaging tests, re-consultation, and physiotherapy, among others [3, 5].

LBP is potentially a long-term health condition characterized by a variable clinical course and multiple interrelated symptomatic episodes [8]. Most LBP episodes have a favourable prognosis, with 90% of patients recovering within 6 weeks [8, 9]. However, LBP recurrences are very common, with 1-year recurrence rates ranging from 33 to 70% [10, 11], of which 40% of people seek health care [11]. Recurrences are the main reason for the consumption of health resources due to LBP [11, 12], as well as for sickness certificates and daily activities limitations. In addition, repeated episodes of recurrence seem to have an increasing duration, work impact and costs, further contributing to the burden of this health condition [5, 13].

By contrast with thousands of studies that assess treatment effects, robust evidence about secondary prevention of LBP remains scarce [14, 15]. Furthermore, studies that analyse specific treatments targeting the symptoms of a current episode of LBP have shown no effects on recurrence rates [14]. A systematic review on this topic suggested that post-discharge exercise programmes seem to be the best strategy with promising results in reducing the risk of LBP recurrence [14]. Likewise, another systematic review with meta-analysis found that exercise alone or in combination with education could reduce 35% and 45% of the risk of an LBP episode, respectively [16]. Despite these results, the same systematic review pointed out that exercise programmes seem to be particularly effective in reducing LBP recurrences in the short to medium term (< 1 year), progressively losing their protective effect over time [16].

Two recent randomized clinical trials seem to corroborate these data, showing that education and exercise programmes can reduce care seeking compared to minimal intervention [17], although they do not meaningfully reduce the risk of LBP recurrence [17, 18]. In both studies, the authors reported low adherence to exercise programmes (consisting mostly of autonomous exercise), which may help to explain the limited effects of exercise on LBP recurrences over time [17, 18]. Considering this issue, it has been argued that exercise programmes should be designed to facilitate the regular and autonomous performance of exercise beyond the intervention period, namely be driven by a theory of behaviour change [14, 19–21]. The use of theory within interventions may provide a greater understanding of the determinants of the target behaviours (i.e., regular exercise) as well as help to identify effective techniques to modify them. Indeed, previous research has suggested that theory-driven interventions are more effective than those that are not, promoting adherence to intervention and maintenance of long-term self-management behaviours such as physical activity [22–24].

On the other hand, previous studies do not integrate comprehensive and long-term assessments of the impact of preventive interventions in real contexts of practice such as primary health care (including their cost-effectiveness) [25] or whether its implementation is possible with sufficient fidelity to preserve its effectiveness. The implementation of innovative practices in new contexts is a complex process that includes multiple potential barriers [26]. The integration of different stakeholders, such as patients, healthcare professionals, or healthcare managers in the implementation process, as well as understanding the context, are critical elements for increasing the potential success and future sustainability of innovative practices [27]. Furthermore, this shared and interactive process is essential to assess effectiveness in context and identify strategies that facilitate and accelerate the knowledge transferability and innovative practices to the clinical practice.

This study seeks to address these issues, describing a hybrid effectiveness-implementation study, which will (1) analyse the effectiveness of a tailored exercise and behavioural change programme and usual care versus usual care alone in the secondary prevention of LBP and (2) evaluate acceptability, feasibility, and determinants of implementation by the different stakeholders, as well as the implementation strategy of the MyBack programme in real context.

## Methods

**Fig. 1:**
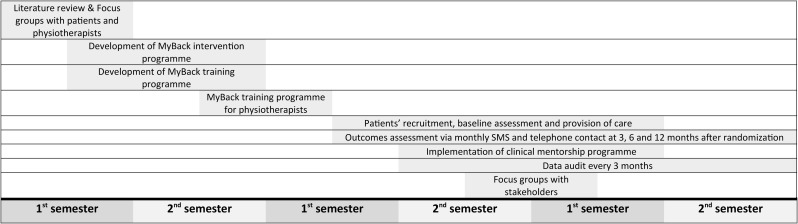
Study timeline

This protocol is reported in accordance with the Standards for Reporting Implementation Studies (StaRI) checklist [28] and was previously register in Clinicaltrials.gov (NCT05841732). The study timeline is shown in Fig. 1.

### Study design and setting

**Fig. 2:**
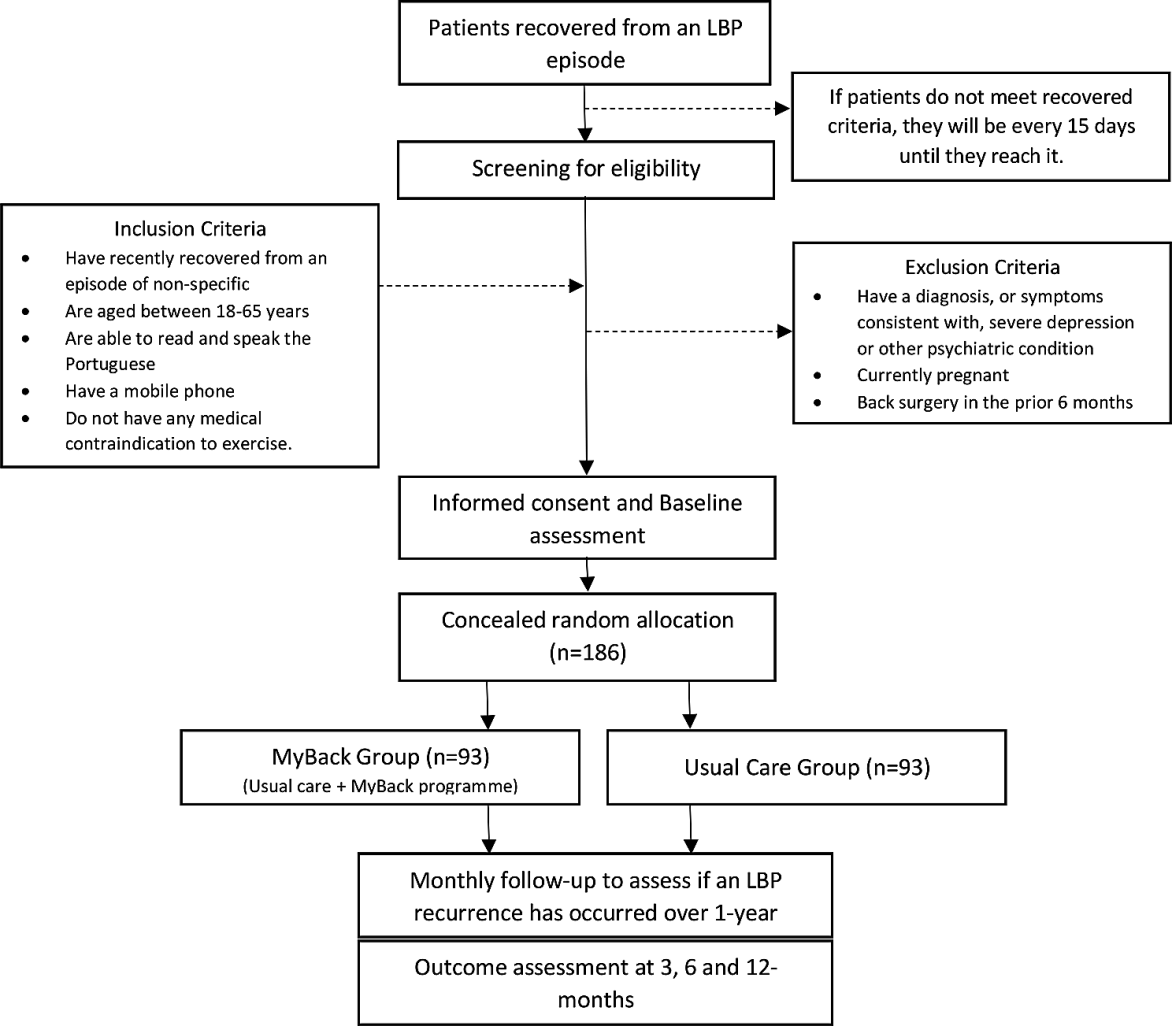
Study design (#Aim I: Effectiveness)

We will conduct a hybrid type I, pragmatic, randomized, controlled, and multicentre study to evaluate the effectiveness and implementation of an intervention programme in the context of primary health care (PHC). The study will be carried out over a period of 3 years in 10 primary health units (HU) located in both urban and rural areas, which provide healthcare (including physiotherapy services) to approximately 950 000 people. All PHCs/HUs partners in the MyBack study have recently implemented an interdisciplinary stratified model of treatment for patients with an episode of LBP [29]. Therefore, the MyBack study will be conducted alongside with the current model of treatment for patients who recovered from the LBP episode in the different partner HU. The MyBack trial design (#Aim I: Effectiveness) is illustrated in Fig. 2.

### Participants

Eligibility criteria will be assessed by trained general practitioners in each partner HU. Participants will be eligible if they: (1) have recently recovered (within the last 3 months) from an episode of non-specific LBP (with or without leg pain and of any duration), defined as pain limited to the region between the lower margins of the 12th rib and the gluteal folds to which it is not possible to attribute a specific anatomical or nociceptive physiopathological cause [30, 31]; (2) are aged between 18 and 65 years; (3) are able to read and speak the Portuguese language; (4) have a mobile phone capable of receiving and sending text messages; (5) and do not have any medical contraindication to exercise. Recovery from an LBP episode is defined as having a pain score of “0” or “1” on a 11-point Numeric Pain Rating Scale (NPRS) for, at least, 30 consecutive days [32]. This means that only participants without pain (or minimal pain – 1/10 NPRS) for at least a month are eligible. Additionally, patients will be excluded if they have a diagnosis, or symptoms consistent with, severe depression or other psychiatric condition, if they are pregnant, if they have undergone back surgery in the prior 6 months, or if their proficiency in Portuguese language is inadequate to complete self-reported outcome measures.

### Recruitment

Potential participants will be recruited from 10 HUs through the collaboration of at least one physiotherapist (PT) from each HU. At the discharge session of the usual treatment programme for an LBP episode, potential participants will receive an information sheet giving details of the MyBack study and will be invited to participate by their PT. Patients who agree to participate will sign the informed consent form and will proceed to a standardized assessment to establish full eligibility. Patients who agree to participate in the study but who have not yet reached the recovery criteria at that time will be contacted every 15 days over a 3-month period, until they reach it. This contact will be made by text message according to the following statement: “On an 11-point scale where “0” means no pain and “10” the worst pain imaginable, in the last month, my back pain was always “0” or “1” (yes / No).

### Baseline assessment

After eligibility criteria have been confirmed, participants will attend a baseline assessment. All participants will complete the baseline assessment on-site that will include a questionnaire of prognostic factors for LBP recurrence and long-term disability [33–35] and specific instruments to assess pain intensity, functional disability, musculoskeletal health, and health-related quality of life (HRQoL). A description of the study measures is presented in Table 1.

**Table 1:** Overview of study measurement

Construct/ Variables	Instrument/ Test	Time point			
		Baseline	Monthly	3, 6 & 12-months	
Demographics	Self-reported questionnaire [33–35]	x			
Clinical		x			
Work Situation**		x	x		
LBP recurrences	The return of LBP with a minimum duration of 24 h, with a pain intensity ≥ 2 on an 11-point numerical scale, preceded by a minimum period of 30 days without pain [32]		x		
Pain intensity**	Numeric Pain Rating Scale (0 to 10) [54, 65]	x	x	x	
Functional Disability**	Roland Morris Disability Scale (0 to 24) [56]	x	x	x	
Musculoskeletal Health (Subdomains: pain severity, physical function, work interference, social interference, sleep, fatigue, emotional health, physical activity, independence, understanding, confidence to self-manage and overall impact)	Musculoskeletal Health Questionnaire (0 to 56) [60, 66]	x		x	
Health-related Quality of Life	EuroQol, 5 dimensions, 5 levels (EQ-5D-5 L) questionnaire (0 to 1) [57]	x		x	
Aerobic capacity*	6-minute walking test [40]	x			
Trunk and lower limb resistance*	Trunk flexor test; Side bridge; Biering-Sörensen test; 60s sit to stand test [41–45]	x			
Trunk and lower limb flexibility*	Sit-and-reach test; Flexion and extension Schober test; Modified Thomas test [46–48]	x			
Motor Control*	Aberrant movement pattern and prone instability test [49, 50]	x			
Health care utilization and LBP Treatments (medical consultations, medication or imaging tests prescribed, referral to other interventions or to other medical specialties or services or sickness certificates) **	Self-reported questionnaire	x	x	x	

*MyBack group only; **Also measured when participants report a LBP recurrence in the screening that occurs every month.

### Randomization and blinding

A randomization scheme will be developed prior to the beginning of the trial, for each HU, by an independent statistician. The randomization will be performed using block randomization (randomly permuted block sizes 4 and 6) in a 1:1 ratio and stratified by HU. According to the randomization scheme, opaque envelopes, sealed and numbered consecutively, indicating the intervention that the participant will receive, will be prepared, and provided to the different local physiotherapists. Immediately after baseline assessment, the PT will open the sequence opaque envelope previously received from a centralized randomization service to allocate the patient to the MyBack (exercise, behaviour change and usual care) group or usual care group. Due to the nature of the study and interventions, it will not be possible to completely blind participants and physiotherapists. To minimise this limitation, it will be explained to participants that the study compares two different approaches for preventing LBP recurrences and participants allocated to usual care group will receive minimal educational intervention. Additionally, researchers responsible for outcome assessments and statistical analysis will remain blinded to group allocation.

### MyBack intervention programme and MyBack training programme

The Behaviour Change Wheel (BCW) Framework [36] will be used to guide the development of the behavioural component of the MyBack intervention programme and the training programme for physiotherapists involved in its implementation. Accordingly, the development process of both programmes followed the multi-staged approach described by Michie, Atkins, and West (2014) [37]: (1) defining the problem in behavioural terms; (2) selecting the target behaviour; (3) specifying the target behaviour; (4) identifying what needs to change; (5) identifying intervention functions; (6) identifying policy categories; (7) selecting behaviour change techniques; and, (8) determining the mode of delivery. These steps will be informed by an extensive literature review on the determinants of adherence to exercise and physical activity programmes, and effectiveness of behavioural change techniques. In addition, data from 4 focus groups will be used to inform the development of the two programmes: two focus groups will be conducted with patients who recently discharged from LBP treatment at the partner HUs in order to identify potential barriers and facilitators for adoption of regular exercise; two focus groups will be conducted with physiotherapists working in the partner HUs in order to identify potential barriers and facilitators for the implementation in real context of the MyBack programme. All determinants identified from the literature review and the focus groups will be analysed by two independent researchers through a deductive content analysis, based on the constructs of the COM-B model and Theoretical Domains Framework (as described in the BCW) [38]. A third researcher will be approached to settle disagreements.

In parallel, an inductive thematic analysis [39] of the focus groups’ data will be conducted to complement the deductive analysis. It is expected that this approach will allow a deeper understanding of the context and the needs of the participants not identified through the BCW codification. Moreover, potential feasibility and acceptability of the MyBack programmes will be also explored through the combination of both analyses.

**Fig. 3:**
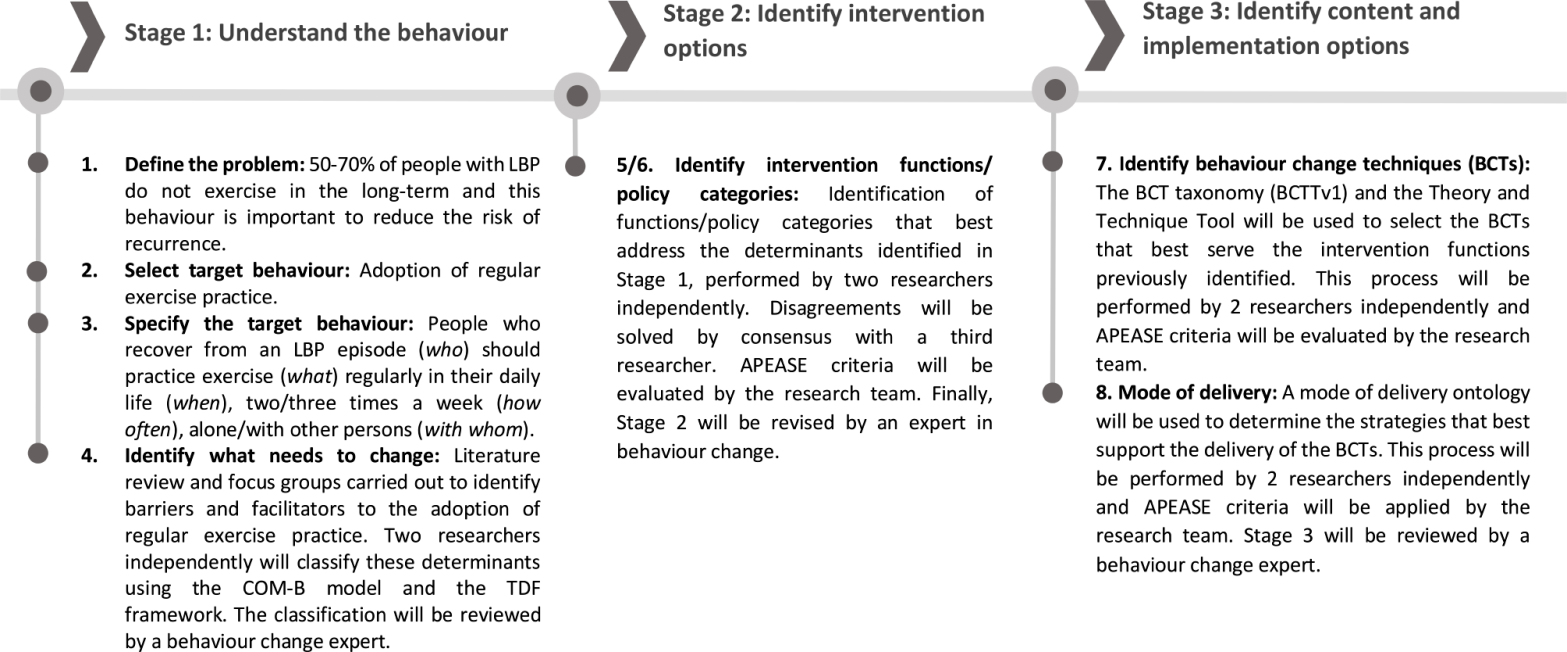
Flow diagram representing the development of the MyBack programmes based on the stages of the behaviour change wheel

Afterwards, the codification of the determinants will be linked to the intervention functions that best address them. Behaviour change techniques and modes of delivery that best tackle the intervention functions will be selected using the BCT taxonomy (BCTTv1) [37] and a mode of delivery ontology [38], respectively. This development process of MyBack programmes will be led by 5 members of the research team and each step will be revised by an external behavioural expert. The remaining team members will participate in consensus workshops on key decisions, applying the APEASE criteria (affordability, practicability, effectiveness and cost-effectiveness, acceptability, side-effects/ safety, and equity) to ensure that decisions are appropriate to the context. At the end, two mapping tables will be produced to inform the delivery of both MyBack programmes, comprising the BCTs and modes of delivery that might be used to target specific barriers related to the target behaviour. This process is illustrated in Fig. 3 and a detailed description of the content of both programmes will be published elsewhere.

### MyBack group

Patients in the MyBack group will participate in a patient-centred, tailored exercise programme informed by a behavioural change approach in addition to receiving usual care in their HU. The MyBack intervention programme will consist of 12 bi-weekly sessions (60 min each) over 6 weeks complemented by 12 exercise sessions to be carried out autonomously by the participants over the following 6 weeks (If the exercise programme includes an aerobic component, a third home-based exercise session will be included).

An initial clinical appointment will be scheduled to assess participants’ physical performance using specific tests, namely: aerobic capacity (6-minute walk test) [40], trunk (trunk flexor test, side bridge, Biering-Sörensen test) [41–44] and lower limb resistance (60s sit to stand test) [45], trunk and lower limb flexibility (sit-and-reach test, flexion and extension Schober test, Modified Thomas test) [46–48] and motor control (aberrant movement pattern and prone instability test) [49, 50]. Based on this assessment and on principles of exercise prescription (FITT-VP principles) [51], a tailored and structured exercise programme will be implemented. Therefore, the exercise component of the MyBack programme may include a variety of types of exercise including aerobic, strength, motor control or flexibility exercises. For example, if changes in motor control are not found, the participant will not perform motor control exercises, and emphasis will be placed on the other altered physical performance components.

All exercise sessions will be complemented with a behavioural change component aimed at promoting the adoption of the exercise programme (supervised and autonomous). This component aims to empower participants to integrate exercise and/or physical activity into their daily lives. Behavioural change component will be also tailored as physiotherapists will adjust content, delivery modes and behavioural change techniques to participant preferences and to individual determinants for target behaviour. Physiotherapists and participants will have access to documentation that will support the implementation of the MyBack exercise programme, including an online patient education package with key messages and BCTs (Physiotherapists) as well as videos supporting its exercise component (Physiotherapists and participants).

#### Usual care group

Participants allocated to the usual care group will be informed that they can access their GP in the usual way (GPs consultation, pain medication, referral for other treatments/ services) and that they should contact their GP if their condition worsens. In addition, they will receive a minimal educational intervention focused on symptom management and promotion of physical activity (“stay active”). This will be delivered in a 15-20-minute face-to-face appointment with their PT. They will be informed that they will be contacted periodically through a text message and by phone so their LBP condition can be monitored.

### Physiotherapist training programme

Intervention providers will be PTs working in the different partner HUs. Before the start of the study, all PTs will participate in online theoretical sessions (10 h) and in a three-day (24 h) practical workshop. The training programme will be focused on exercise testing and prescription in patients at risk of LBP recurrence and the development of necessary skills to promote the target behaviour. These include the use of BCTs targeting specific barriers to facilitate the adoption of regular exercise practice and the use of motivational interviewing principles to guide the whole intervention. As described above, PTs training programme will be developed using BCW framework to support PTs’ adoption of these new practices. It is expected the use of different modes of delivery, including role-playing activities, discussion of case scenarios and practical demonstrations.

Additionally, a clinical mentorship programme and evaluation of fidelity of the delivery will take place throughout the implementation. PTs will have ongoing support provided by two members of the research team, who will be available to discuss their clinical cases and clarify any doubts that may arise. Monthly, peer feedback and review of clinical cases will be provided to PTs by the MyBack team. PTs will also complete case report forms (CRF) detailing the interventions provided following the items of the Consensus on Exercise Reporting Template (CERT) checklist [52]. CRF will be reviewed regularly by two elements of research team to determine if the programme has been delivered as intended.

### Data collection and outcomes

#### #Aim I: effectiveness

Two trained researchers, blind to the patients’ group, will be responsible for the organization and data collection process. Every 30 days after the date of randomization, and over a 1-year period, the participants of both groups will receive a text message (SMS API (twilio.com)) with a yes/no question regarding LBP recurrence (In the last month did you have pain in your back, with an intensity equal or greater than 2, on a scale where “0” means no pain and “10” the worst pain imaginable and lasting at least 24 h?). This question is based on the definition of LBP recurrences: “the return of LBP with a minimum duration of 24 hours, with a pain intensity ≥ 2 on an 11-point numerical scale, preceded by a minimum period of 30 days without pain” [53]. All text messages will be sent and received through a specific SMS platform. In the presence of a LBP recurrence, the researchers will contact the participant by telephone to answer to a brief survey concerning the date of the recurrence and its impact (if the episode was severe enough to limit activity and/ or to cause care seeking), and to fulfil the self-reporting instruments (Table 1).

In the absence of a LBP recurrence, the researchers will contact the participants at three fixed data points: 3, 6 and 12 months after baseline, to fulfil the NPRS, Roland-Morris Disability Questionnaire (RMDQ), Musculoskeletal Health Questionnaire (MSK-HQ) and EuroQol (EQ-5D-3 L) (Table 1). The estimated patient’s burden to answer all the questionnaires is approximately 15 min. In addition, the participants’ medical records will be reviewed every 3 months. Data on any additional medical consultations, medication or imaging tests prescribed, referral to other interventions or to other medical specialities or services or sickness certificates will be recorded on the patient’s CRF.

Participants will remain on the trial unless they choose to withdraw consent, or they fail to answer three consecutive follow-up text messages. To prevent withdrawals and when a participant does not answer to 3 SMS text message or is not available at fixed follow-up time points, a maximum of three additional attempts will be made by telephone call.

The primary outcome of the study will be the risk of LBP recurrence, i.e. the percentage of participants experiencing one or more episodes of LBP recurrence within a 12-month follow-up period. Secondary outcomes include pain intensity, functional disability, musculoskeletal health and HRQoL. Pain intensity will be measured by a NPRS (11-point version). The NPRS has proven to be valid and reliable in patients with LBP [54, 55]. Disability status and HRQoL will be evaluated through the Portuguese versions of the Roland Morris Disability Questionnaire (RMDQ) and the EQ-5D, 5 L. Both self-reported measurement instruments have been cross-culturally validated to Portuguese language and showed good to excellent psychometric properties [56, 57]. The Portuguese version of the Arthritis Research UK Musculoskeletal Health Questionnaire (MSK-HQ) will be used to measured musculoskeletal health and 12 related subdomains (pain severity, physical function, work interference, social interference, sleep, fatigue, emotional health, physical activity, independence, understanding, confidence to self-manage and overall impact). The original and Portuguese version of MSK-HQ showed adequate psychometric properties in people with musculoskeletal pain [58–60].

Costs data will be collected to both groups from medical health records, and they will consist of all back pain-related recourse used within primary health care by each participant. For the MyBack group, the costs related to the MyBack programme (costs per session; costs of training physiotherapists; costs of materials and transport) will also be collected.

#### #Aim II: implementation

A mixed methods approach will be implemented in parallel with the effectiveness study and will help to understand the study processes or underlying mechanisms in relation to context, setting, professionals and patients. The exploration of feasibility, acceptability, fidelity, and contextual determinants of change is expected to provide knowledge that will improve the success of the implementation of different study tasks, such as the delivery of a training programme for physiotherapists or the provision of care. This stage will be informed by the Behaviour Change Wheel guidance [37]. Additionally, this knowledge will provide an opportunity for future triangulation of data obtained from other study stages and data. For this purpose, information about the perceptions and experiences of all stakeholders, namely patients, health professionals and HUs coordinators, and participation data will be collected. This phase will be informed by the Medical Research Council (MRC) guidance for the development and evaluation of complex interventions and the conceptual model of implementation research proposed by Proctor et al. (2011) [61] as well as the RE-AIM Planning and Evaluation framework [62] to evaluate implementation outcomes and its potential to scale-out across primary care.

Focus groups with patients, health professionals and HUs coordinators, will be carried throughout the implementation of MyBack study. Focus groups with HUs coordinators and health professionals will be focused on the exploration of their perceptions regarding the feasibility, acceptability, and fidelity of delivery of the intervention, considering the competences needed as well as the resources available. Information about additional facilitators and barriers will be collected in order to identify contextual determinants of change that were not identified prior to implementation and should be addressed by the research team. Focus groups with patients will be held as they acknowledge them as active participants in health interventions. These focus groups will explore the fidelity of receipt, that is, fidelity with which the content of the intervention is received by patients, as well as the acceptability of the intervention, barriers, and facilitators for the maintenance of exercise in the long term.

A total of 5 focus groups, including a minimum of 6 and maximum of 8 participants per group, will be carried out throughout the implementation of the MyBack study: 1 with HUs coordinators from the 3 PCC; 2 with health professionals from the 3 PCCs; 2 with patients. Thus, a minimum of 30 participants (6 HUs coordinators; 12 health professionals; 12 patients) are expected to be purposively selected from those who are going to participate in the study, by receiving or delivering MyBack programme or coordinating services for its implementation. To be included, patients are expected to have attended to a minimum of 12 treatment sessions. The focus groups will have an expected duration of 90 min per group and will be carried out by videoconference. All focus groups will be held by 2 researchers (CC; SD) with previous experience in facilitating focus group discussions. The discussions will be based on a semi-structured interview schedule previously developed and tested in pilot interviews. All focus groups will be audio-recorded and transcribed verbatim.

Additionally, study enrolment and participation data will be considered to evaluate the reach of the project in each practice by tracking the proportion of eligible patients referred to the study. Patients` adherence, defined as the completion of 12 or more treatment sessions, will be measured for all participants randomized into the MyBack group.

### Sample size (Aim I: effectiveness)

The sample size was estimated to the primary outcome (risk of recurrence) and based on recurrence rates described in previous studies. Assuming a recurrence proportion of 40% in 12 months [11], an alpha value of 0.05 and a power of 80%, a sample size of 81 participants per group will be necessary to detect a difference of, at least, 20% between the two groups (relative risk = 0.50) [14, 63]. The choice of the minimal clinically relevant difference was informed by a previous systematic review [14] and was considered realistic considering the study hypothesis. Based on previous studies carried out in the same settings [64], it is expected a dropout rate of 5% during the MyBack programme and a 10% loss of participants during follow-up. Therefore, the total sample size to be recruited will be 186 participants, 93 for each group.

### Data handling and analysis plan

A data management plan will be developed in advance entailing data collection procedures, missing data management, coding, and entry of variables in the electronic database. An independent researcher blind to the intervention groups will be responsible for transferring the patients’ data to the electronic database. All data will be stored in a coded format and, thus, unreadable without the codebook previously developed and kept securely under principal researcher responsibility. A data audit will be conducted every 3 months. Discrepancies between original data and database, and missing data, will be checked and corrected. Any missing values will be accounted for using multiple imputation methods.

#### #Aim I: effectiveness

To assess the effectiveness of the MyBack programme, a quantitative analysis of primary and secondary outcomes will be made using multivariable regression models. All quantitative analysis will be conducted by a statistician who will be masked to the intervention groups. The analyses will follow the intention-to-treat principle. Between-group differences for the primary outcome (risk of LBP recurrence) will be analysed using relative risk reduction (95%IC and significance level of *p* ≤ 0.05). Secondarily, survival curves for each group will be described and compared (log-rank test) using the days from randomization to the first episode of recurrence. Scores of pain intensity, disability, musculoskeletal health and HRQoL will be assessed for outliers, normality and homogeneity. In the case of parametric distribution, the differences between the two groups over time will be analysed using two-way repeated measures ANOVA. If significant, the Bonferroni post-hoc test will be used. In addition, a subgroup analysis will be performed according to potential differences between the participants who adhered or did not adhere to the experimental intervention.

The cost-effectiveness analysis will be developed from the perspective of the health care provider. All costs will be calculated for each participant and indexed in Euros for the reference year 2023. Quality-adjusted life years (QALYs) will be calculated with the EQ-5D, 5 L. Between-group differences for costs, QALYs and years of life gained will be analysed following the non-parametric bootstrap method. A cost-effectiveness analysis will be conducted using the incremental cost per QALY ratio.

#### #Aim II: implementation

Qualitative data analysis will be performed in a similar way for all focus groups. The focus group discussions will be transcribed verbatim and anonymized by a researcher and then checked by a second researcher. An inductive thematic analysis will be used to achieve an in-depth data description through the identification, analysis and presentation of the relevant themes and subtheme. This analysis will follow the six phases described by Braun and Clarke (2006) [39]. In parallel, a deductive content analysis will be performed using a coding matrix based on the TDF domains and the COM-B components. The deductive analysis will be guided by the study’s aims of identifying barriers and facilitators to implementation of MyBack programme. Both analyses will be carried out independently by two researchers. A third researcher was approached to settle disagreements.

### Ethical issues and study supervisory group

Ethical approval was granted by the Ethics Committee of Regional Health Administration of Alentejo Central. The study will be conducted in compliance with the Declaration of Helsinki (1964 and amendments).

All potential participants will receive a participant information sheet with detailed information about “MyBack” study as well as contacts from research team for additional information, doubts or claims throughout the study. Those who agree to participate will be asked to give their written informed consent. All participants will be invited to participate voluntarily, and no economic retribution will be provided. No risks or discomforts are expected, since procedures for data collection will be based in self-reported measures, information from clinical records, text messages answers (yes/ no) or audio-records, and treatment will follow usual practice and current scientific recommendations. Nevertheless, safety reporting procedures will be in place to ensure any expected and unexpected serious adverse events, which could be deemed to be related to the study. Participants will be also informed about the possibility to withdraw at any moment. In this case, data associated with the participant will be erased.

All collected data will be securely stored with restrict access from the research team. Data will be kept in a locked file in a research building. All data will be preserved for a maximum of 5 years after the end of the study and then will be destroyed. Personal data will be managed following the privacy policy of leader institution and the General Data Protection Regulation (GDPR) established by the European Union diploma 2016/679.

A committee (Study Supervisory Group) formed by 2 patients, 4 health professionals and 2 researchers will be formed to continually assess the quality standards of the study and scientific and technical deliverables from an end user and informed stakeholder perspective. They will provide suggestions for improvement and will actively participate in the analysis, write-up, and subsequent dissemination of the study findings.

## Discussion

### Potential impact in clinical practice and research

Considering the worldwide impact of LBP on health systems and its high recurrences rate, it has been suggested that the development of effective prevention strategies has the potential to reduce the burden associated with this health condition. Thereby, MyBack is the first known exercise programme complemented by a behavior change approach designed aiming the secondary prevention of LBP. The MyBack programme will also be designed to allow for tailoring of both the exercise and behavioral change components, thus responding to the unique needs and characteristics of each patient at risk of LBP recurrence. Therefore, it is expected that data on the effectiveness, cost-effectiveness, and implementation of the MyBack programme may contribute to improve health care delivery in primary health care, contributing to direct and indirect costs reduction for patients and the health system in the secondary prevention of LBP.

Additionally, the MyBack study addresses important limitations identified in previous studies. It is expected that the population of this study is representative of LBP patients seeking primary health care, as well as the sample size allows a robust and accurate data analysis. Also, the development of a theory-driven intervention to prevent recurrences has been suggested as an optimal way to promote population-level health behaviour change in the real-world context.

### Methodological challenges

This project has some methodological challenges that should be addressed to guarantee its feasibility. First, MyBack comprises a 12-week intervention for asymptomatic LBP patients, which may increase the risk of dropouts. Likewise, long-term follow-up may also compromise participants collaboration in the study. These aspects highlight the need to develop strategies that promote participant adherence, both during the intervention and during the follow-up period. Additionally, despite the number of partners HUs seems to be sufficient to safeguard the recruitment of the sample, MyBack study requires a large sample size.

Finally, it is not possible to exclude the risk that the intervention is not adequately provided by the collaborator PTs as the MyBack project involves a significant change in care delivery. The implementation of a clinical mentorship programme following the training programme aims to support and expand the time for the development of PTs expertise in implementing this change. Although efforts will be made to ensure that PTs are adequately trained and supported throughout the study, it can be challenging to ensure intervention fidelity and strategies will be developed to control this issue.

### Dissemination plan

The outputs and results of this study will be disseminated through manuscript publications in prominent scientific journals and presentations at national and international conferences. All members of the research team will be involved and will actively collaborate in the planning and development of actions to disseminate the findings of the MyBack project.

## Data Availability

Not applicable.
